# A deficit of detoxification enzymes: pesticide sensitivity and environmental response in the honeybee

**DOI:** 10.1111/j.1365-2583.2006.00672.x

**Published:** 2006-10

**Authors:** C Claudianos, H Ranson, R M Johnson, S Biswas, M A Schuler, M R Berenbaum, R Feyereisen, J G Oakeshott

**Affiliations:** * Research School of Biological Sciences, Australian National University Canberra, ACT, Australia; † Commonwealth Scientific and Industrial Research Organization (CSIRO) Entomology Canberra, ACT, Australia; ‡ Liverpool School of Tropical Medicine Liverpool, UK; § Department of Entomology, University of Illinois Urbana, IL, USA; ¶ Department of Cell and Structural Biology, University of Illinois Urbana, IL, USA; ** Institut National de la Recherche Agronomique (INRA) and Université de Nice Sophia Antipolis, Centre de Recherche de Sophia Antipolis Sophia Antipolis, France

**Keywords:** honeybee, glutathione-S-transferase, cytochrome P450 monooxygenase, esterase, insecticide resistance

## Abstract

The honeybee genome has substantially fewer protein coding genes (≈ 11 000 genes) than *Drosophila melanogaster* (≈ 13 500) and *Anopheles gambiae* (≈ 14 000). Some of the most marked differences occur in three superfamilies encoding xenobiotic detoxifying enzymes. Specifically there are only about half as many glutathione-S-transferases (GSTs), cytochrome P450 monooxygenases (P450s) and carboxyl/cholinesterases (CCEs) in the honeybee. This includes 10-fold or greater shortfalls in the numbers of Delta and Epsilon GSTs and CYP4 P450s, members of which clades have been recurrently associated with insecticide resistance in other species. These shortfalls may contribute to the sensitivity of the honeybee to insecticides. On the other hand there are some recent radiations in CYP6, CYP9 and certain CCE clades in *A. mellifera* that could be associated with the evolution of the hormonal and chemosensory processes underpinning its highly organized eusociality.

## Introduction

One of the most striking features of the *Apis mellifera* genome is that it contains significantly fewer annotated genes than other insect genomes so far sequenced. It contains only about 11 000 protein coding genes (Honey Bee Genome Sequencing Consortium, 2006), whereas the fruit fly *Drosophila melanogaster* has about 13 500 and the malarial mosquito *Anopheles gambiae* has about 14 000 (Holt *et al*., 2002). Another invertebrate, the free-living nematode *Caenorhabditis elegans*, has even more, over 19 000 (Consortium, 1998). Why might the honeybee be so depauperate genetically? Is it because of some overarching property of the honeybee's genetic system, such as its haplodiploidy, or does it reflect more specific tailoring of its genetic architecture to its unique and highly specialized life history and control of its environment? Has its highly organized eusociality and the limited exposure of pre-adults and reproductives to the external environment obviated the need for certain functions involved with environmental interactions? In this paper we address these questions by comparing the complements of three protein superfamilies heavily involved in environmental interactions across *A. mellifera*, *D. melanogaster* and *An. gambiae*.

The three superfamilies we analyse are the glutathione-S-transferases (GSTs), cytochrome P450s (P450s) and carboxyl/cholinesterases (CCEs). While some members of all three superfamilies, particularly the P450s and CCEs, are known to have other functions, for example in various biosyntheses and signalling processes, genes from several clades in these superfamilies have been directly implicated in the detoxification of xenobiotics. In other insects these three superfamilies are heavily involved in insecticide metabolism and they contribute the great majority of mutations conferring metabolic resistance to chemical insecticides (Feyereisen, 2005; Oakeshott *et al*., 2005a; Ranson & Hemingway, 2005). There is also evidence for the involvement of P450s and CCEs at least in insecticide metabolism in honeybees (Gilbert & Wilkinson, 1974; Yu *et al*., 1984; Pilling *et al*., 1995; Suchail *et al*., 2004).

The honeybee is unusually sensitive to a range of chemical insecticides (Johansen & Kleinschmidt, 1972; Atkins, 1975; Devillers *et al*., 2002; Stefanidou *et al*., 2003). Foraging bees may encounter lethal insecticide levels when pollinating agricultural fields or foraging in residential settings and additional mortality can occur when contaminated nectar and pollen are brought back to the hive. Classes of pesticide with representatives that are considered highly toxic (i.e. direct contact toxicity up to days after application to crops, with a kill rate of over 1000 per hive per day) include carbamates, organophosphates, synthetic pyrethroids, chlorinated cylcodienes and chloronicotines. Incidents of bee colony losses of unexplained origin have led to recent restrictions on fipronil and imidacloprid use in France by application of the ‘precautionary principle’. Major metabolic (or target site) resistance mutations have not yet been described in this species, although there are reports of race-based differences in sensitivity to pyrethroid insecticides (Danka *et al*., 1986; Elzen *et al*., 2003).

## Results

### Glutathione-S-transferases

Just 10 putatively functional GST genes have been identified in *A. mellifera*, which is far fewer than the numbers in this superfamily in *D. melanogaster* and *An. gambiae*. Furthermore, the distribution of these GSTs across the six classes found in other insects differs substantially from that of the mosquito and fruit fly. The honeybee is particularly depauperate in the insect-specific Delta and Epsilon classes but the Sigma class is actually larger in this species (Table 1). Half of the honeybee GSTs are represented by ESTs (Table 2).

**Table 1:** Number and class distribution of insect GSTs, P450s and CCEs from the genomes of *D. melanogaster* (fly); *An. gambiae* (mosquito) and *A. mellifera* (honeybee), see also Figs 1, 2 and 4

Superfamily	Class/clade/family	Fly	Mosquito	Honeybee
GST	Delta	11	12	1
	Epsilon	14	8	0
	Omega	5	1	1
	Sigma	1	1	4
	Theta	4	2	1
	Zeta	2	1	1
	unknown	0	3	0
	Microsomal*	1	3	2
	Subtotal	38	31	10
P450	CYP4 clade	32	45	4
	Families	*4*, *311*–*313*, *316*, *318*	*4*, *325*	*4*
	CYP3 clade	36	42	28
	Families	*6*, *9*, *28*, *308*–*310*, *317*	*6*, *9*, *329*	*6*, *9*, *336*
	CYP2 clade	6	10	8
	Families	*18*, *303*–*307*	*15, 303*–*307*	*15*, *18*, *303*, *305*–*307*, *342*, *343*
	Mitochondrial CYPs	11	9	6
	Families	*12*, *49*, *301*, *302*, *314*, *315*	*12*, *49*, *301*, *302*, *314*, *315*	*301*, *302*, *314*, *315*, *334*
	Subtotal	85	106	46
CCE	Dietary class			
	A clade	0	0	8
	B clade	2	14	0
	C clade	11	2	0
	Pheromone/hormone processing class			
	D clade	3	0	1
	E clade	3	4	3
	F clade	2	4	0
	G clade	0	4	1
	Neuro/developmental class			
	H clade	4	9	0
	I clade	2	2	2
	J clade	1	2	2
	K clade	1	1	1
	L clade	4	5	5
	M clade	2	2	1
	Subtotal	35	51	24
Total		157	188	80

**Figure 1:**
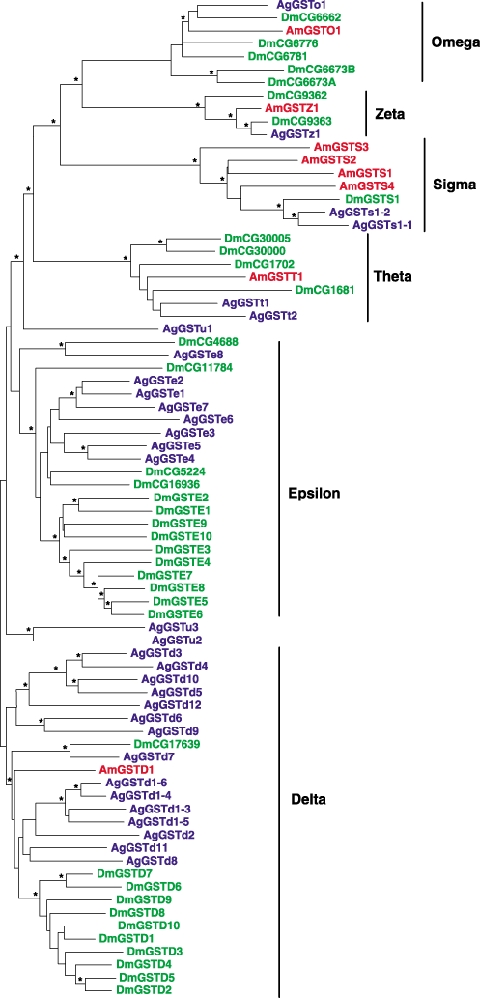
Unrooted distance neighbour-joining tree showing phylogenetic relationships of the predicted GST proteins of *A. mellifera* (shown in red), *D. melanogaster* (green) and *An. gambiae* (blue). Radiations corresponding to the recognized Delta, Epsilon, Omega, Sigma, Theta and Zeta classes of GST are indicated. Alternative splice variants of the *An. gambiae* GSTS1 and GSTD1 sequences are designated by a hyphenated Arabic number. Distance bootstrap values of greater than 70% (500 replicates) are indicated at the relevant nodes by an asterisk (*).

* Microsomal GSTs not used in analysis of Fig. 1.

**Table 2:** Gene name, annotation, location, functional assignment and expression data for *Apis mellifera* GSTs, P450s and CCEs. Unless otherwise specified all ESTs are from female (worker) bees

Gene name	BeeBase identifier*	Location: (linkage group/chromosome)†	Putative function (sequence orthology)	EST (expression)
GSTs				
GSTS1	GB16959	Un.1306		Brain
GSTS2		Un.1306		Brain
GSTS3	GB19254	Un.898		Brain
GSTS4	GB14372	4		
GSTD1	GB18045	15		Brain, antennae, various tissues
GSTO1	GB11466	1		Brain
GSTT1	GB12047	Un.336		
GSTZ1	GB17672	5		
GST mic1‡	GB12371	2		Larval caste
GST mic2‡	GB10566	1		
P450s				
6AQ1	GB15409	12		Antennae
6AR1	GB17588	5		
6AS1	GB16899	Un.16938		
6AS10	GB14594	13		
6AS11	GB11027	13		
6AS12	GB12136	Un.7647		
6AS13	GB17831	Un.16945		Brain, adult
6AS14	GB19113	13		
6AS15	GB14913	Un.7743		
6AS16P	GB12885	Un 16945	Putative pseudogene	
6AS17	GB10668	13		
6AS18	GB11620	13		
6AS2	GB19197	Un.7649		Male antennae, brain
6AS3	GB15681	Un.7649		
6AS4	GB15793	Un.7647		
6AS5	GB17434	13		
6AS7	GB18052	13		Brain, adult, whole body
6AS8	GB11754	13		
6AS9P	GB18672	13	Putative pseudogene	
6BC1	GB10466	1		
6BD1	GB19306	11		
6BE1	GB14612	Un.9058		
9P1	GB14836	14		
9P2	GB19055	14		
9Q1	GB19820	14		Brain
9Q2	GB17793	14		Brain
9Q3	GB19967	14		Brain
9R1	GB16803	14		Brain
9S1	GB13748	14		
336A1	GB19797	2		Brain, adult
4AV1	GB16698	Un.7150		
	GB18743			
4AZ1	GB10905	Un.12437		
4AA1	GB17784	Un 8535		
4G11	GB11973	16		Brain, whole body, larval
15A1	GB15634	7	Methyl farnesoate epoxidase (JH biosynthesis)	
18A1	GB14343	13		
303A1	GB18872	Un.7777		
305D1	GB11943	7		Brain, adult
306A1	GB12311	13	Ecdysteroid 25-hydroxylase	Brain
307B1	GB18019	14		
342A1	GB10856	8		
343A1	GB14915	Un.8401–8402		
49A1	GB17396	Un.6830		
301A1	GB11406	Un.6830		
302A1	GB15545 (5′)	Un.17961	Ecdysteroid 22-hydroxylase	
	GB14974 (3′)	Un.14595		
314A1	GB13998	5	Ecdysteroid 20-hydroxylase	
315A1	GB16447	Un.7658	Ecdysteroid 2-hydroxylase	
334A1	GB12608	9		
CCEs				
–	GB16342	1	Odourant degrading	
–	GB14476	15	Odourant degrading	
–	GB19866	14		Brain
–	GB10854	14		
–	GB15030	7		
–	GB13591	7		
–	GB13602	11	OP-resistance	Brain
–	GB11064	Un.234		Antennae, brain
–	GB16889	7	Integument esterase	Brain
–	GB15327	3	Pheromone degrading	Male antennae, brain, whole body, various stages
–	GB10820	3		
–	GB11403	12		Brain
–	GB18660	Un.2	JHE hormone degrading	
–	GB15536	10		
–	GB18901	Un.435		
AChE-1	GB18414	11	Cholinesterase	Brain
AChE-2	GB14873	8	Cholinesterase	Brain
gli	GB12309	Un.112	Gliotactin (gli)	
NLG-1	GB18720	9	Neuroligin	
NLG-2	GB10066	1	Neuroligin	
NLG-5	GB13939	9	Neuroligin	Brain
NLG-3	GB18290	9	Neuroligin	Brain
NLG-4	GB18836	9	Neuroligin	
nrt	GB19830	14	neurotactin	Embryo

* BeeBase sequences predicted from genome assembly Amel 3.

† Un. prefix denotes unplaced contig.

‡ Microsomal GSTs not included in analysis of Fig. 1.

Two partial GSTs have also been identified in the genome sequence data set. Transcripts from these putative GST genes have not been detected, so it is not clear whether they are truncated pseudogenes or full genes that are simply not completely covered in Version 3.0 of the data set. There is also a further GST-like sequence in the data set (GB10031-PA) that has very high identity to a proteobacterial GST and most probably represents a bacterial contaminant or a GST from an endosymbiont.

### The insect specific classes of glutathione-S-transferase

Two classes of GST, the Delta and Epsilon classes, are found uniquely in insects. Both of these classes are represented by a minimum of eight genes in the mosquito and fruit fly and the members of these gene families are largely the product of local gene duplications. The independent radiation of these gene families in *An. gambiae* and *D. melanogaster* and the functional verification of the role of a subset of these enzymes in xenobiotic detoxification suggest that they are important in the adaptation of insects to environmental selection pressures (Ranson *et al*., 2002). Indeed one of the best characterized functions of these insect GST classes is their role in insecticide detoxification. A small subset of the Delta and Epsilon class insect GSTs can catalyse the dehydrochlorination of the organochlorine insecticide DDT (Tang & Tu, 1994; Ranson *et al*., 2001; Lumjuan *et al*., 2005) and other members of the insect-specific GST classes metabolize organophosphate insecticides (Huang *et al*., 1998; Wei *et al*., 2001). Whereas other classes of insect GSTs have been proposed to play a secondary role in protection against insecticides, for example by ameliorating the effects of oxidative stress, all those GSTs directly implicated in insecticide metabolism to date belong to the Delta or Epsilon classes. Hence, the complete absence of the Epsilon class in the honeybee and the presence of only a single Delta class GST may partially account for the extreme sensitivity of this species to certain insecticides.

The *A. mellifera* Delta GST GSTD1 is the only member of this superfamily for which tissue distribution data are available. This enzyme is widely distributed in bee tissues, having been detected in the brain, reproductive tissue, thorax and abdomen by reverse transcription–polymerase chain reaction (Collins *et al*., 2004; Corona *et al*., 2005) but interestingly an EST derived from GSTD1 has also been detected in a cDNA library from female antennae (http://www.tigr.org). A Delta class GST in the moth *Manduca sexta* is specifically located in antennae and involved in odour degradation (Rogers *et al*., 1999). Although the ubiquitous distribution of *A. mellifera* GSTD1 precludes a specific role in the olfactory response, it may serve a general function in protecting the bee from exposure to harmful volatile xenobiotics. This enzyme has also been postulated to play a role in protection against oxidative stress (Collins *et al*., 2004). It is found at higher levels in the spermatheca of mated vs. unmated queens and the suggestion is that this GST aids in the protection of sperm from oxidants. Functional characterization of this enzyme is needed to verify this prediction.

### The Sigma glutathione-S-transferase class

In contrast to the scarcity of Delta and Epsilon GSTs in the honeybee, the species has more members of the Sigma class (four genes vs. a single Sigma GST gene in the Diptera). In the house fly and fruit fly the Sigma GSTs possess a proline/alanine-rich N-terminal extension, which interacts with Troponin H in the insect flight muscle, which has led to the suggestion that these enzymes play an important structural role in this tissue (Clayton *et al*., 1998). However, the butterfly *Papilio multicaudatus* has multiple Sigma GSTs that lack this extension and are postulated to play a catalytic role, in the metabolism of plant allelochemicals (MRB, MAS unpublished data). The mosquito Sigma GST, and all four of the Sigma GSTs in *A. mellifera*, also lack this hydrophobic extension. In general, Sigma GSTs show low levels of activity with the typical GST substrates but they have a high affinity for the lipid peroxidation product 4-hydroxynonenal and their localization in metabolically active tissues in flies, such as the flight muscles, has been suggested to be instrumental in protecting these tissues from by-products of oxidative stress (Singh *et al*., 2001). Three of the four honeybee Sigma GSTs are represented by ESTs and all 21 ESTs so far recorded for this GST class were detected in cDNA libraries constructed from bee brains (Table 2). EST data can provide only a snapshot of tissue distribution but it is intriguing to question why these GSTs are so well represented in this tissue.

*A. mellifera* GSTS1 and GSTS2 represent the only case of local duplication within the honeybee GST superfamily. Both of these genes are located in close proximity on a linkage group of unknown chromosomal location. The two additional Sigma GSTs, GSTS3 and GSTS4, are dispersed in the genome but all four GSTs show conservation in the intron position. The introns within the Sigma GSTs in *An. gambiae* and *D. melanogaster* are also found at the same position. In *An. gambiae* the single Sigma GST gene is alternatively spliced with one of the transcripts containing four exons (with introns in identical positions to those in GSTS1, -S3 and -S4 in *A. mellifera*) and the second transcript lacking the same intron as is missing in *A. mellifera* GSTS2 (Ding *et al*., 2003).

### Other classes of glutathione-S-transferases

The remaining GST classes represented in the honeybee, the Omega, Theta and Zeta classes, are ubiquitously distributed in nature, suggesting that they play key roles in metabolic processes as opposed to the more general detoxification role of the Delta and Epsilon GSTs. This hypothesis is supported by the demonstration of the catalysis of tyrosine and phenylalanine degradation by the Zeta class GSTs (Board *et al*., 1997) and the importance of Omega GSTs in the removal of S-thiol adducts from proteins (Board *et al*., 2000). Both of these classes are represented by a single gene in *A. mellifera* and secure 1 : 1 : 1 orthologues can be identified between these honeybee GSTs and GST genes in *An. gambiae* and *D. melanogaster* (Fig. 1). *D. melanogaster* also contains an additional Zeta class GST, the product of local gene duplication. A comparison of the intron positions within the Zeta genes from all three insect species reveals that the mosquito and fruit fly have a conserved intron toward the 5′ end of the coding sequence that is absent from the honeybee. However, the *A. mellifera* GSTZ1 has a total of four unique introns, contrasting with the single intron in the *An. gambiae* GSTZ1 and the two introns in the orthologue of this gene in *D. melanogaster*.

The Omega GST in *A. mellifera* is represented by a single EST in the brain (although one of the two partial GST genes described above has been tentatively assigned to this class). As is found in the Zeta GSTs, the Omega class is also expanded in *D. melanogaster* compared with the honeybee or mosquito (Fig. 1) and the reasons for this expansion are presently unclear. A similar situation is observed for the Theta class but in this case, multiple genes are found in both *D. melanogaster* and *An. gambiae* as compared with the single gene in *A. mellifera.* The Theta class, although also widely distributed in mammals, plants and invertebrates, has not yet been attributed a particular function in the metabolism of endogenous substrates, and it is possible that the expansion of this GST class in the Diptera serves to enhance their capacity to detoxify xenobiotics.

### The microsomal glutathione-S-transferases

The presence of two genes putatively encoding microsomal GSTs in the honeybee is mentioned for completeness (Table 1). Although the microsomal GSTs play a similar role to the cytosolic enzymes in general detoxification reactions and protection against oxidative stress, they bear no structural resemblance to the cytosolic GSTs and have a different genetic origin (Hayes *et al*., 2005). The microsomal GSTs are membrane-bound and, in humans at least, assemble to form homotrimeric proteins (Schmidt-Krey *et al*., 1999).

### Cytochrome P450s

With 46 putatively functional P450 genes, the honeybee genome in Version 3.0 encodes far fewer P450s than the genomes of *D. melanogaster* (85 genes) and *An. gambiae* (106) (http://P450.antibes.inra.fr; Table 1). Some of the 46 are not complete in Version 3.0 but in three cases, sequence has been completed independently by polymerase chain reaction/cDNA sequencing [CYP314A1 (DQ244074), CYP4G11 (DQ244075) and CYP6AS5 (DQ232888); RMJ and MRB unpublished results]. The sequences of five others (CYP4AA1, CYP6BC1, CYP302A1, CYP303A1, CYP343A1) are informed guesses, because they are not found on contiguous pieces of assembled genomic DNA. Only 13 of the honeybee P450 genes are currently represented by ESTs (Table 2).

As with the GSTs, the honeybee genome also yields a very different frequency distribution of P450s across the major clades of this superfamily from those found in the two Diptera (Table 1). Four phylogenetically distinct clades can be distinguished: the mitochondrial P450s, and the CYP2, -3 and -4 clades named after the prominent vertebrate members of these clades. The CYP3 clade, of which human CYP3A4 is the best known member, includes the large CYP6 and CYP9 families of insects. The CYP3 and CYP4 clades are both abundant in the dipteran repertoires and each accounts for about 40% of their P450 complements. Most of the overall scarcity in the honeybee is due to a massive shortfall in CYP4 numbers (four sequences only), whereas the CYP6 and CYP9 families are only slightly less numerous. The size of the CYP2 clade, which in insects comprises a variety of single-member families, is maintained in the honeybee (eight compared with six and 10 in the fruit fly and mosquito). The mitochondrial P450s are significantly reduced by the total absence of CYP12 sequences, whereas the other mitochondrial P450s, mostly 1 : 1 : 1 orthologues in the three species, are maintained. The net effect is that CYP6 family members account for fully 43% of the honeybee P450s.

It is difficult to predict the specific functions of most of the honeybee P450s simply from their sequence similarities. A single amino acid change can significantly alter the metabolic capabilities of a P450 (Lindberg & Negishi, 1989; Wen *et al*., 2005) and even relatively similar P450s can have widely different capacities for xenobiotic metabolism (Li *et al*., 2004). Members of the CYP4, -6, -9 and -12 radiations have all been implicated in environmental response/detoxifying functions in other insects, with members of the CYP4 and CYP6 groups most commonly involved in insecticide metabolism and resistance (Berenbaum, 2002; Feyereisen, 2005). On the other hand several enzymes from the CYP2 and mitochondrial CYP families have recently been implicated in essential hormone biosynthesis and further correlations between function and phylogenetic classes may emerge over time. In this context, it seems likely that the absence of so many CYP4s and all the CYP12s in *A. mellifera* reflects a loss of environmental response and detoxifying capability, while the retention of many CYP2 clade and the remaining mitochondrial P450 sequences would be consistent with a large role of these P450s in the metabolism of hormones required widely across the Insecta. Why the CYP6s should so predominate the *A. mellifera* P450 complement is presently mysterious.

### The mitochondrial and CYP2 clades

**Figure 2:**
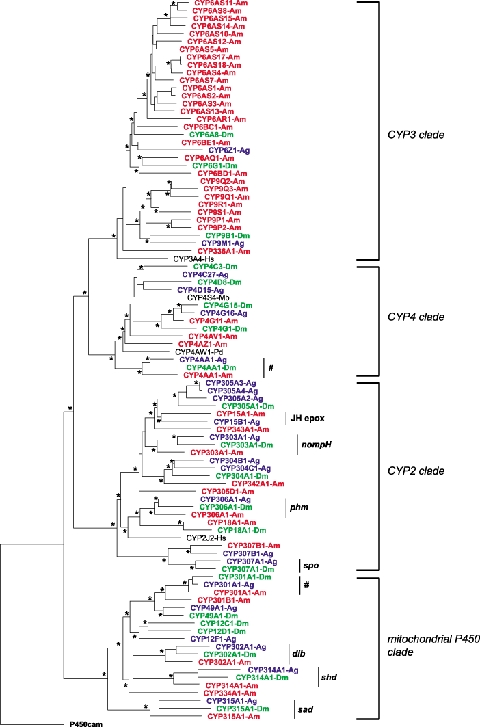
Neighbour-joining phylogeny of the P450 sequences from the honeybee (in red) with selected P450s from *D. melanogaster* (Dm – green) and *An. gambiae* (Ag – blue). The outgroup (P450cam, the camphor hydroxylase from *Pseudomonas putida*), human CYP2J2 and CYP3A4, and two other P450s mentioned in the text (CYP4S4 from *Mamestra brassicae* and CYP4AW1 fom *Phyllopertha diversa*, both possibly involved in odour or pheromone clearance) are marked in blue. Orthologues of the *D. melanogaster Halloween* gene products involved in ecdysteroid biosynthesis (phm, spo, dib, shd, sad) and of the nompH gene product are marked on the right, as well as the CYP15 sequences orthologous to the juvenile hormone epoxidase identified in the cockroach. Two additional series of orthologues (CYP4AA1 and CYP301A1) of unknown function are marked by a hatch (#). The great diversity of the fruit fly and mosquito CYP3 and CYP4 clades and mitochondrial CYP12 family (see Table 1) is represented by just a few members for clarity of the tree. Inclusion of the other members does not alter the overall topology of the tree. Distance bootstrap values of greater than 50% (1000 replicates) are indicated at the relevant nodes by an asterisk (*).

Several honeybee sequences in these series show close sequence similarities, in some cases precise orthologies, with *D. melanogaster* enzymes with specific roles in the biosynthesis of hormones (Fig. 2). It is highly likely that they have similar roles in *A. mellifera*. Those likely to be involved in ecdysteroid biosynthesis include the mitochondrial CYP302A1, -314A1 and -315A1 P450s, which are orthologues of the enzymes encoded by the *D. melanogaster* Halloween genes *dib*, *shd* and *sad*; these encode the steroid 22-, 20- and 2-hydroxylases, respectively (Gilbert, 2004). These three mitochondrial P450s as well as the CYP301A1 sequence, of unknown function, are 1 : 1 : 1 orthologues in the honeybee, mosquito and fruit fly.

**Figure 3:**
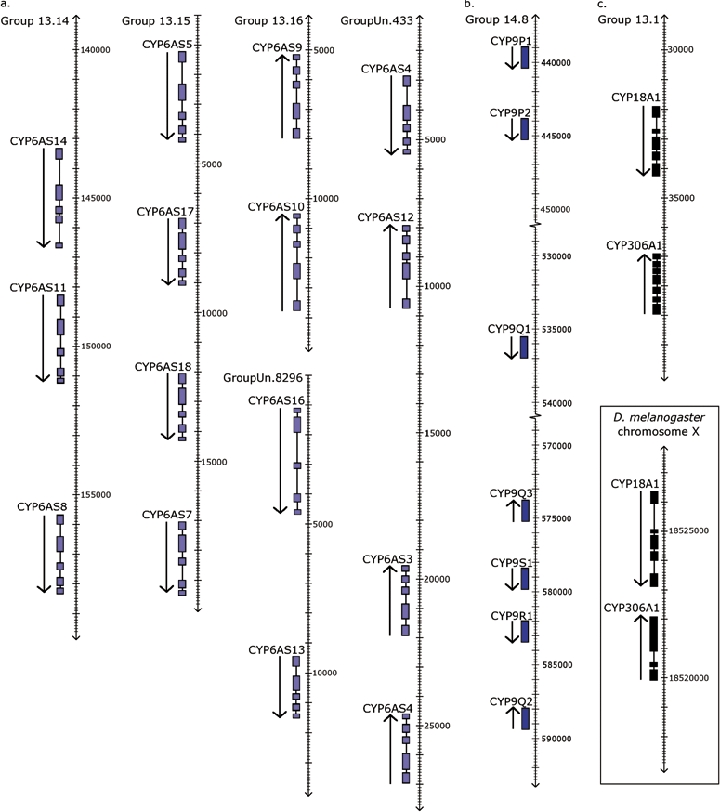
Genomic regions encoding clusters of P450 genes in *A. mellifera*. Boxes correspond to exons, lines represent introns, and arrows indicate gene orientation. (A) Nine of 17 CYP6AS subfamily genes map to chromosome 13 on three adjacent scaffolds and six are clustered with at least one other CYP6AS subfamily member on two unassigned scaffolds. (B) All eight intronless CYP9 family genes map to a 16 kb region on chromosome 14. (C) The tight linkage of CYP18A1 and CYP306A1 on chromosome 13 is a case of possible microsynteny (albeit a different gene orientation) with the region of the *D. melanogaster* genome containing their putative orthologues.

There are also closely similar microsomal CYP306A1 and -307A1 P450s that are orthologues of the enzymes encoded by the *D. melanogaster phm* and *spo* genes; *phm* encodes an ecdysteroid 25-hydroxylase (Niwa *et al*., 2004) and *spo* is of as yet unknown function. *Spo* has a Halloween-type developmental phenotype, and is expressed in the prothoracic glands of the silk moth *Bombyx mori* at times of high ecdysone titre, but not in the prothoracic glands cells in the *Drosophila* ring gland (Namiki *et al*., 2005). The *D. melanogaster* orthologue of CYP18A1 is ecdysone-regulated (Bassett *et al*., 1997) and possibly encodes an ecdysteroid 26-hydroxylase/oxidase. Evidence supporting this speculation is the high level of sequence conservation (36% amino acid similarity) between CYP306A1 (a 25-hydroxylase) and CYP18A1, and the conserved clustered organization of the CYP306A1 and CYP18A1 genes between *D. melanogaster* and the honeybee (Fig. 3). Interestingly, however, CYP18A1 has no orthologue in *An. gambiae*, and there is no direct empirical evidence that ecdysteroids are metabolized by 26-hydroxylation/oxidation in any of the species.

The only other *A. mellifera* enzyme in this series to which a specific function can be confidently ascribed is CYP15A1, which is clearly the orthologue of juvenile hormone epoxidase from the cockroach *Diploptera punctata* (Helvig *et al*., 2004a) and *An. gambiae*. CYP303A1 is worthy of note because it has very close 1 : 1 : 1 orthologues in the fruit fly and mosquito. The *D. melanogaster* CYP303A1 gene is expressed selectively in sensory bristles and is encoded by the *nompH* gene whose mutants have mechano- and chemosensory defects (Willingham & Keil, 2004).

### The depauperate CYP4 clade

The CYP4 clade is a highly diversified group of enzymes in insects; CYP4s in other insects have been implicated in both pesticide metabolism and chemical communication. CYP4G8 is overexpressed in pyrethroid-resistant strains of *Helicoverpa armigera* (Pittendrigh *et al*., 1997). CYP4C27 in *An. gambiae* is overexpressed in a DDT-resistant strain (David *et al*., 2005) and CYP4G19 expression in *Blattella germanica* is correlated with pyrethroid resistance (Pridgeon *et al*., 2003). Several CYP4 genes are overexpressed in pesticide resistant *Culex pipiens* and *Diabrotica virgifera* (Scharf *et al*., 2001; Shen *et al*., 2003). Expression of CYP4AW1 in the scarabaeid beetle *Phyllopertha diversa* is antenna-specific and its inhibition interferes with pheromone perception, suggesting a role in pheromone degradation (Maibeche-Coisne *et al*., 2004). In the noctuid moth *Mamestra brassicae* four P450s, CYP4L4, CYP4S4, CYP4G20 and CYP9A13, are expressed in sensilla trichodea of the antennae (Maibeche-Coisne *et al*., 2002, 2005). The expression of CYP4S4 is limited to these sensory units of the antennae that are tuned to the detection of odourants and sex pheromones, whereas the other three are also expressed in other olfactory or gustatory structures.

Specific functions have not been determined empirically for any of the four CYP4s surviving in the honeybee, although there are some useful indicators in some features of their sequences. CYP4AA1 has true 1 : 1 : 1 orthologues in the two other species, and the presence of an EST from prothoracic glands in *Bombyx mori* suggests that it plays a part in ecdysteroid synthesis. CYP4G11 was the first known (partial) P450 sequence from the honeybee (Tares *et al*., 2000), which is not surprising in view of the large number of ESTs found for this P450. Many ESTs are also found in *Drosophila* for its putative functional homologues, CYP4G1 (Tijet *et al*., 2001) and CYP4G15, and in *An. gambiae* for CYP4G16 and 4G17. CYP4G11 is more highly expressed in workers than queens (Evans & Wheeler, 2001). Members of the insect CYP4G subfamily are also notable for an unusual long insertion between helices F and G, and a nontraditional N-terminal sequence. These features indicate that these proteins may have an unusual subcellular distribution, perhaps linked to function. The other two honeybee CYP4s, CYP4AV1 and CYP4AZ1, are paralogues of CYP4s that appear to be related to lipid metabolism (Feyereisen, 2005), including CYP4AB1 and CYP4AB2 that are overexpressed in workers of the fire ant *Solenopsis invicta* (Liu & Zhang, 2004).

### Recent radiations of CYP6s and CYP9s

Whereas both the CYP6 and CYP9 families in the CYP3 clade appear to be well-represented in the honeybee genome, the close proximity of these sequences on chromosomes 13 and 14 and the conserved number of introns suggest that 15 of the CYP6 sequences and all CYP9 sequences are the products of relatively recent gene duplication events (Fig. 3). Members of the CYP6AS subfamily all share four introns of exactly conserved position and there are at least three CYP6AS pseudogenes. Similarly, all honeybee CYP9s are intronless, suggesting rapid and recent duplications of an initially retrotransposed gene.

Consistent with the clustered organization and conservation of intron positions, there are also quite high levels of amino acid and nucleotide similarity both among the CYP6s and among all the CYP9s above. Amino acid identities/similarities are all at least 30/50% among these CYP6s and 34/56% among the CYP9s. Differences in silent nucleotide sites (K_s_, the number of silent substitutions per silent site) are as little as 0.135 and 0.235 for several terminal bifurcations in these radiations (CYP6AS1–2; CYP9P1–2, respectively; Fig. 2) and only about 1.87 even for the most distant comparisons (CYP6AS1–CYP6AR1) within them. As we will show below (see Discussion), these values strongly suggest that most of these two radiations have occurred since the *A. cerana-mellifera* group of species diverged from *A. florea*, *A. dorsata* and *A. laboriosa*.

Clusters of CYP6 and CYP9 genes are found in other insects and some code for P450s involved in pyrethroid resistance (Feyereisen, 2005 for a review). For example, CYP6Z1 is overexpressed in pyrethroid resistant *An. gambiae* and is clustered with 20 other P450s in the genome (Nikou *et al*., 2003). CYP6D1 and CYP6D3, both associated with pyrethroid resistance in *Musca domestica*, are in a similar cluster (Kasai & Scott, 2001). Similarly, CYP6G1, associated with DDT and neonicotinoid resistance in *D. melanogaster*, is part of a three gene cluster (Daborn *et al*., 2002). CYP321A1 of *Helicoverpa zea*, a member of the CYP3 clade, metabolizes cypermethrin (as well as the plant toxins xanthotoxin and angelicin) when produced in a baculovirus expression system (Sasabe *et al*., 2004).

It would seem at face value that the honeybee may have ample numbers of CYP6s and CYP9s to deal with a range of potential substrates and on this basis it might be argued that these classes of P450s could generate significant environmental response and pesticide detoxification capability. Whether in honeybees these P450s do contribute to xenobiotic detoxification awaits empirical functional analyses, although they seem good candidates to account for such P450 metabolism of pesticides as has been attributed to the species by work to date (Pilling *et al*., 1995; Suchail *et al*., 2004). It will be particularly interesting to see whether the limited sequence divergence among the recent radiations of the CYP6s and CYP9s also limits their functional diversification. Coupled with the paucity of CYP4s and the absence of CYP12s, any lack of functional diversity among these classes could severely limit the potential of honeybees to respond to diverse chemical challenges.

### P450s and chemical communication

Honeybees are heavily dependent on semiochemical signalling, both for interindividual communication and for tracing food resources through floral scents, and this dependence is reflected elsewhere in the genome in the substantial expansion of gene families encoding odourant receptors (Honey Bee Genome Sequencing Consortium, 2006). Although a sensitive response to plant allelochemicals is probably needed by many other bees, the extraordinary polylectic foraging behaviour of *A. mellifera* relative to any other insect pollinators (Schmidt & Johnson, 1984) suggests that this species encounters and processes a much broader range of floral signals than do other bees. As well, much of the complexity of the interindividual communication shown by honeybees may have evolved as an integral part of their highly advanced eusociality as the genus diverged from other genera in the family. The pheromone repertoire of *A. mellifera* is among the most extensive known in invertebrates. Pheromones are involved in their foraging ontogeny, colony defence, queen attendance, nestmate recognition, queen cell acceptance, forage choice behaviour and brood care. Moreover most of these signals are multicomponent mixtures (Pankiw, 2004). Morphological and anatomical features of *A. mellifera*'s olfactory system, including 10-fold greater numbers of drone olfactory poreplate sensilla, relative to its congener *A. florea*, also suggest more complex pheromone processing and greater sensitivity even within the genus (Brockmann & Bruckner, 2001).

As principal participants in insect biosynthetic pathways and significant players also in semiochemical reception, P450s might be expected to be more numerous in insects such as honeybees whose metabolism involves relatively high levels of chemical complexity (Wilson, 1971). Clearly, in the case of the honeybee, they are not. On the other hand it may be that a significant proportion of those P450s that have been retained in honeybees are involved in either pheromone biosynthesis or the peri-receptor phenomena involved in the receipt and transmission of semiochemical signals (Vogt, 2003). Just as one example involving pheromone synthesis, the worker- and queen-selective mandibular acids are biosynthesized from stearic acid by ω and ω-1 hydroxylations, respectively, both reactions that are likely to be catalysed by P450 enzymes (Plettner *et al*., 1998). However, empirical evidence for the involvement of specific P450s in such processes is currently limited. Fatty acid hydroxylations are notoriously catalysed by CYP4 enzymes in vertebrates, but to date the only insect enzyme shown to catalyse fatty acid ω-1 hydroxylation is CYP6A8 from *D. melanogaster* (Helvig *et al*., 2004b). Enzymes involved in insect pheromone metabolism are now being studied much more intensively than in the past, which should help resolve this paradox of diverse needs for P450 capability and relatively limited availability of P450 diversity.

### Carboxyl/cholinesterases

The *D. melanogaster* and *An. gambiae* genomes contain 35 and 51 members of the carboxyl/cholinesterase superfamily, respectively, organized in about 13 major clades and three major classes (Ranson *et al*., 2002; Oakeshott *et al*., 2005a). The most ancient class contains proteins implicated in neuro/developmental functions and all but one component clade, the acetylcholinesterases (AChEs), are catalytically inactive. Several of the clades in this class have *C. elegans* and vertebrate members, whereas neither of the other two classes is represented in vertebrates and there is just a single *C. elegans* representative in one of them. The second class contains proteins whose sequences suggest that they are secreted, generally catalytically active enzymes. Functional data on a few suggest relatively specific functions in hormone and pheromone processing. The third class contains catalytically active enzymes, generally intracellular but with a range of microsomal, mitochondrial and cytosolic localizations. Most esterases implicated in insecticide detoxification and metabolic resistance belong to this third class, with a few also in the second.

The *A. mellifera* sequence to date contains just 24 carboxyl/cholinesterases, so this group shows the same trend for fewer members as do the other two gene families (Table 1). All 24 sequences appear to be functional genes, in that they lack disabling frameshifts, premature stops and the like, but 5′- and 3′-termini could not always be unambiguously identified (see below). The sequences are dispersed across 19 locations and at least 11 chromosomes (Table 2). Unlike the situation for *D. melanogaster* and *An. gambiae* there are no large eight to 10 member clusters of genes and very few pairs with high sequence similarity suggestive of recent duplication activity. Indeed just nine of the *A. mellifera* genes are colocated in four small clusters (of two, two, two and three members) and in all but one case nearest relatives show less than 40% amino acid identity (see below). Thus, in marked contrast to the situation in the other two species, there is no evidence for very recently duplicated genes in transition to new functions.

**Figure 4:**
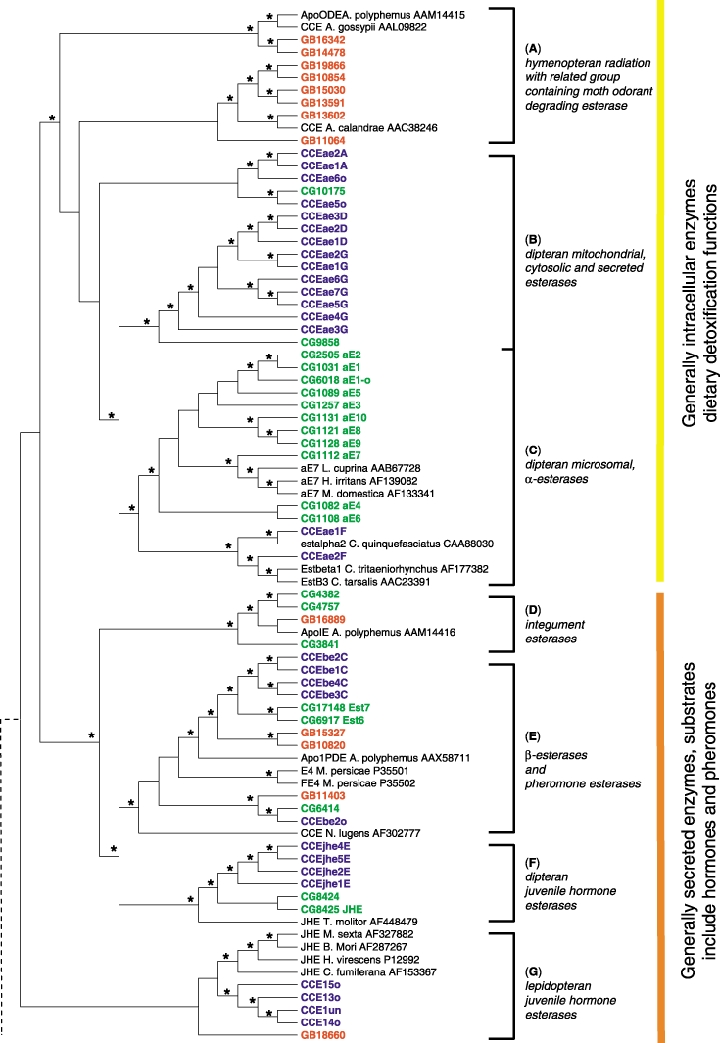

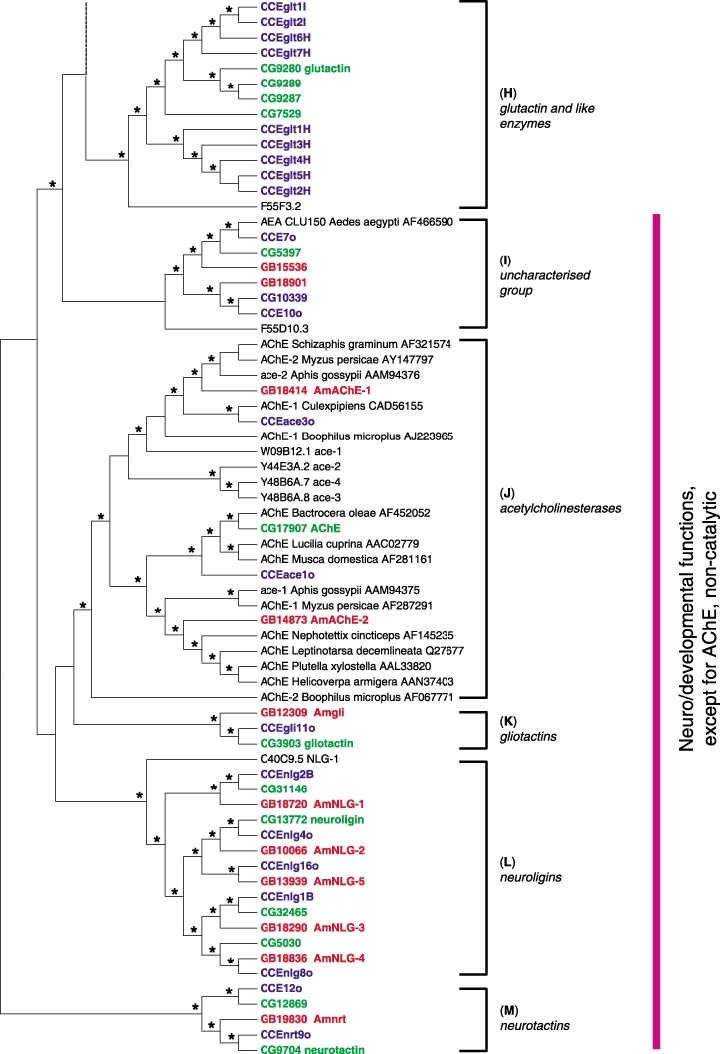
Unrooted distance neighbour-joining tree showing a phylogeny of carboxyl/cholinesterase (CCE) proteins. Sequences from *A. mellifera*, *D. melanogaster* and *An. gambiae* genomes (red, green and blue, respectively) are shown with other previously characterized CCE sequences, NCBI accession number provided (see also Tables 1 and 2). Sequences were aligned using Clustal W followed by some minor corrections to conform to known structural features of CCEs and did not include N- and C-terminal extensions typical of many neuro/developmental members of this family. The resultant ≈ 530 amino acid alignment, spans a region equivalent to residues 65–558 of *D. melanogaster* AChE (CG17907), was analysed with MEGA3.1 (Kumar *et al*., 2004) using the neighbour-joining method with pair-wise deletion of gaps/missing data and the PAM 001 matrix substitution model to construct a phylogenetic tree. The tree (represented as a cladogram) was split into two connecting subtrees corresponding to metabolic enzymes (part A) and neuro/developmental proteins (part B) for ease of viewing. An asterisk (*) indicates nodes with greater than 50% resampling frequency (1000 bootstrap replications). Boundaries for the three major classes and 13 major clades are indicated on the right. Clade boundaries generally agree with those shown in Oakeshott *et al*. (2005a) although the addition of the *A. mellifera* data enhanced resolution among the dietary/detoxification clades A–C and one uncharacterized neuro/developmental clade (I).

Addition of the *A. mellifera* sequences and one recently published moth pheromone esterase to the CCE data set produces a phylogeny (Fig. 4) with broadly similar topology to that of Oakeshott *et al*. (2005a), although there are some minor changes to clade resolution (see below and footnotes to Fig. 4). The honeybee CCEs occur in 10 of the 13 major clades and all three classes evident in the other two species. However, as with the GSTs and P450s, there are large differences between it and the other two species in the representation in individual clades and the deficit of CCEs compared with the other species is highly uneven across the phylogeny.

### Neuro/developmental carboxyl/cholinesterases

Aside from AChE, these molecules are generally noncatalytic adhesive proteins involved in cell–cell interactions. Many of the encoding genes are notably large, up to 250 kb, interrupted by numerous and sometimes large introns. *A. mellifera* has 11 members of the neuro/developmental class (clades I–M in Fig. 4), very comparable with the 10 in *D. melanogaster* and 12 in *An. gambiae*, and there is also a very high level of 1 : 1 : 1 orthology across the three species*.* Sequence identities are generally 35–40% and intron positions (e.g. six of 10–12 introns in neuroligins) are well conserved across orthologues.

Like the other two species, the honeybee has one putative gliotactin and two putative neurotactins. It also has two representatives in the other lineage of catalytically inactive proteins with as yet unknown functions (clade I). Like *An. gambiae* (Ranson *et al*., 2002; Weill *et al*., 2002) it has two AChE genes, *D. melanogaster* (and apparently other higher Diptera) being unusual among the Insecta in having a single AChE (Russell *et al*., 2004). It remains to be determined which *A. mellifera* AChE functions in synaptic processing of acetylcholine; the one characterized to date (GB14873; (Shapira *et al*., 2001) sits in the subclade containing *Drosophila* AChE and the non-neurological AChE of two-AChE insects (Harel *et al*., 2000); Fig. 4). Target site resistance to organophosphate and carbamate insecticides, which in other species is almost always encoded by mutations in the neurological AChE (Russell *et al*., 2004; Oakeshott *et al*., 2005b), has not been recorded in *A. mellifera*.

**Figure 5:**
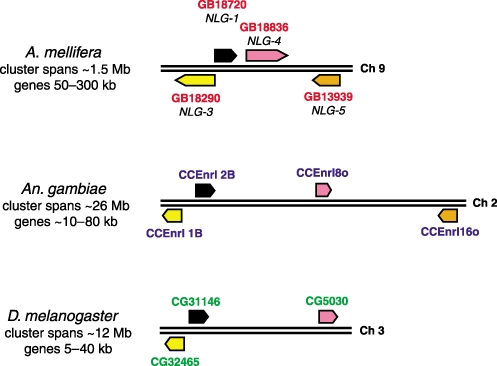
Comparison of the physical locations of honeybee neuroligin genes predicted from the Version 3.0 assembly of the Honeybee Genome Project with orthologues from *D. melanogaster* and *An. gambiae* showing high levels of microsynteny.

The honeybee has five presumptive neuroligins that all share structural features found in the well characterized vertebrate neuroligins. These highly conserved transmembrane molecules are pioneer adhesive proteins involved in formation and specification of synapses in the brain (Dean & Dresbach, 2005). There is support from brain EST expression for GB18290 and GB13939 (Table 2) and we have similarly confirmed adult brain expression for all five honeybee neuroligins (SB, RJ Russell, JGO and CC unpublished data). Among these, three tightly clustered neuroligin genes show remarkable conservation of microsynteny among *A. mellifera*, *An. gambiae* and *D. melanogaster* (Fig. 5). Also notable is the head-to-head organization of NLG-1 (GB18290) and NRL-3 (GB18720), which are less than 2 kb apart in *A. mellifera*, albeit somewhat further apart in the other two species. The honeybee organization at least raises the possibility of some sharing of 5′ regulatory regions, and/or coregulation.

### Carboxyl/cholinesterases involved in hormone and pheromone processing

Whereas *D. melanogaster* has 11 members of the secreted (generally) catalytic class (clades D–H in Fig. 4), and *An. gambiae* has 23, *A. mellifera* has only five. All five of the latter have standard secretion signals and the sequence motifs characteristic of catalytic activity that their placement in this class would predict. They sit in three of the five clades in this class.

One of the clades missing in the honeybee (H), containing the noncatalytic glutactin protein, is likely to pre-date the Insecta as it also contains a *C. elegans* sequence. Therefore, its absence in *A. mellifera* can be attributed to the loss of an ancestral clade in the bee rather than the gain of derived sequences in the other species. Glutactin is the only member of the clade for which there is any functional information; it is abundant in basement membrane sites in the envelope of the central nervous system in *D. melanogaster* and is inferred to play a part in intercellular ordering and adhesion (Darboux *et al*., 1996; Grisaru *et al*., 1999). Whether and how this function is discharged in honeybee remains to be determined.

The other clade of secreted enzymes missing in the honeybee (F) contains a functionally validated juvenile hormone esterase (JHE) from *D. melanogaster*, and putative JHEs from *An. gambiae* and a coleopteran. The best candidate for JHE in *A. mellifera* in fact sits in another clade (G) that contains all the known lepidopteran JHEs. Apart from this phylogenetic location, there is also some more specific sequence data to suggest that this honeybee sequence (GB18660) is indeed JHE. Thus the functionally validated JHEs of other species generally have three basic contact residues in an amphipathic helix located elsewhere in the active site (Thomas *et al*., 1999; Campbell *et al*., 2001; Oakeshott *et al*., 2005a); GB18660 has two of these (alignment not shown). All functionally validated JHEs have a GQSAG nucleophilic elbow motif in their active site, whereas GB18660 does not, albeit no other putative CCE enzyme of *A. mellifera* does either. However, the GHSAG motif found in GB18660 has been recorded in some candidate JHEs in, for example, *An. gambiae*. Interestingly, GB18660 has a nonconsensus GGK signature in another part of its active site, the oxyanion hole (which accommodates an oxyanion intermediate of the carboxylesterase reaction) but *in vitro* mutagenesis of other CCEs shows that basic substitutions in the oxyanion hole, including Lys, are compatible with carboxylesterase activity (Schopfer *et al*., 2004). Also noteworthy, the improvement in tree resolution achieved with the addition of the *A. mellifera* data suggests that the lepidopteran/*A. mellifera* JHE clade (G) is the ancestral JHE condition, with the dipteran/coleopteran JHEs in Clade F being an independent, convergent evolution of this activity.

The other four honeybee members of the secreted catalytic class of clades sit in two clades for which the available functional evidence also suggests roles in hormone or pheromone signalling. One sits in a small clade (D) including a moth integumental CCE implicated in pheromone processing (Ishida & Leal, 2002) and the other three sit in a larger clade (E) containing one validated sex pheromone esterase from the moth *Antheraea polyphemus* (Ishida & Leal, 2005) and the esterase 6 enzyme of *D. melanogaster*, which exercises a different signalling function involved in reproductive behaviour (Oakeshott *et al*., 2005a). Significantly there is also some indirect functional data for one of the three honeybee members of this clade (GB15327); specifically, it has been shown to correspond to the D-AP1 protein that is preferentially expressed in drone antennae (Kamikouchi *et al*., 2004). This evidence suggests to us that the four honeybee members of these two clades are good candidates for hormone and pheromone signalling roles.

Also of interest with respect to D-AP1 is that it has been recovered from brain and other EST libraries (Table 2) and, most unusually, it not only has a conventional amino terminal secretion signal but also has a consensus endoplasmic reticulum (ER) retention signal at its carboxy terminus. The latter of course is usually associated with microsomal proteins, albeit there are precedents for them in secreted proteins, where they appear to function in holding the protein on the ER until some signal triggers their release (Ren *et al*., 2003; Lee *et al*., 2004; Davis *et al*., 2006).

We must also note that there is an alternative annotation of the D-AP1 sequence in the NCBI database as a JHE (AAU81605). We do not know the basis for this assignment but we are reluctant to accept it at this point. Our reasons are its lack of any of the characteristic JHE motifs (see above), its relative abundance in EST libraries (the lepidopteran and *D. melanogaster* JHEs have very tightly regulated expression peaks and have not been recorded even in the very extensive EST databases for these species; http://www.tigr.org/tdb/tgi), its demonstrated concentration in male antennae (where JHE is not expected), the lack of any other insect JHEs in this clade, and the presence of another honeybee sequence with at least some of the characteristic JHE motifs in a clade containing other JHEs.

Whichever is the physiological JHE in *A. mellifera*, it is perhaps surprising that the honeybee genome has just five CCEs in this class. This is the class where we might expect CCEs involved in semiochemical reception to sit. However, while a high proportion of the key molecules are indeed esters, in fact many of the pheromones at least may be beyond the substrate range of many CCEs because the CCEs characterized biochemically from a wide range of species typically act on substrates with bulky alcohol but small acid groups (Arpigny & Jaeger, 1999; Fojan *et al*., 2000). In contrast, most of the esters found in the pheromone repertoire of honeybees have large acid groups and small alcohol moieties and as such would usually be subject to hydrolysis by lipases (Slessor *et al*., 2005). Indeed most of the pheromone esters are methyl or ethyl esters of long chain fatty acids (Slessor *et al*., 2005). It is not out of the question that some CCEs could hydrolyse esters with relatively large acid groups; JH has an acid group with an 11 carbon chain and some synthetic pyrethroid insecticides that are metabolized by CCEs have acid groups of comparable bulk (Heidari *et al*., 2005). However, the fatty acid chains in the fatty acyl ester pheromones are typically 16–20 carbons long. Of the honeybee pheromones thus far characterized perhaps the one most likely to fall within the scope of CCE hydrolysis might be the alarm pheromone component isopentyl acetate (Slessor *et al*., 2005).

On the other hand many floral scents are comprised of esters with shorter acyl groups (Knudsen *et al*., 1993) and it may be that some members of this class of CCEs are involved in the detection of these compounds, and associated food resources.

### Carboxyl/cholinesterases involved in xenobiotic metabolism

This class (clades A–C) contains 13 *D. melanogaster*, 16 *An. gambiae* and eight *A. mellifera* sequences. All of the latter sit in clade A, so clades B and C remain dipteran-specific radiations. In fact all eight appear in a single subclade whose only other member to date is another hymenopteran sequence, from a parasitic wasp, suggesting the possibility that this subclade may also be an order-specific radiation.

Indeed, this latter radiation may be the most recent major addition to the CCE superfamily in the honeybee lineage; relative to other honeybee CCEs, all eight of the *A. mellifera* sequences are quite closely related (36–71% amino acid identity, whereas pair-wise identities among the rest of the honeybee CCEs are generally below 35%). By comparison with some of the recent radiations of CCEs in *D. melanogaster* and *An. gambiae*, however, the honeybee radiation still shows substantial divergence; for example, there are many pair-wise identities among the CCEs of the latter two species that exceed 70% (Oakeshott *et al*., 2005a). Also, although four of the genes in the honeybee radiation are located in two, two-gene clusters, the most parsimonious reconstruction of the radiation still invokes three single gene translocations to distant sites and just two of the classical adjacent, head-to-tail single gene duplications that predominate in the recent radiations of *D. melanogaster* and *An. gambiae* (Ranson *et al*., 2002; Oakeshott *et al*., 2005a). Again, this difference would be consistent with a relatively greater age for the honeybee radiation.

N- and C-termini could not be predicted unambiguously for some of the *A. mellifera* clade A enzymes but those that could be confidently assigned adhered to a general trend toward microsomal localization apparent in several of the *An. gambiae* and *D. melanogaster* members of this clade. Some deviation from the consensus C-terminal KDEL or KKxx ER retrieval or retention signals is apparent in GB13602 (KNKL) and GB13591 (ERNL). However, not all the *Drosophila* or mosquito members of the clade are microsomal either, some having cytosolic or mitochondrial localizations.

Three of the clade A honeybee sequences have highly unusual signatures in one essential active site motif, the oxyanion hole. Compared with the GGG/A consensus seen through most of the CCE superfamily, GB13591 has DGA and GB19866 and GB10854 have EGA. We know of no empirical data on the effects of such substitutions in the first position of the oxyanion hole but *in vitro* mutagenesis of other CCEs has shown that substitutions to His or acidic residues in the third position abolish carboxylester hydrolysis (Heidari *et al*., 2004; Schopfer *et al*., 2004). Remarkably, some of the substitutions with Asp or His in these positions are associated with the acquisition of limited OP hydrolase activity. This acquisition has been seen in both natural OP resistance mutations in higher Diptera and in synthetic esterase mutants tested in the laboratory (Lockridge *et al*., 1997; Newcomb *et al*., 1997; Claudianos *et al*., 1999; Heidari *et al*., 2004; Schopfer *et al*., 2004). There is thus a theoretical possibility that either the DGA or EGA substitutions in the three honeybee CCEs may confer some enhanced tolerance for OPs, although any such effect would seem likely to be small, given the unusually high sensitivity to OPs consistently reported for honeybees (see Introduction). In fact, we suspect that none of these three honeybee enzymes with nonconsensus oxyanion holes have appreciable OP hydrolase or carboxylesterase activities, which would even further reduce the number of *A. mellifera* sequences with potential to act in conventional CCE detoxification roles.

Intriguingly, the CCE from the parasitic wasp that colocalizes to this honeybee radiation has been implicated in OP insecticide resistance (Zhu *et al*., 1999a,b). It has a GGG oxyanion hole and retains carboxylesterase activity but it also has a Trp Gly substitution in the acyl pocket of its active site that provides a limited level of OP hydrolase activity. Substitutions of the corresponding Trp to other small residues such as Leu and Ser in Clade B enzymes also confer this activity (Campbell *et al*., 1998; Claudianos *et al*., 2001; Heidari *et al*., 2004). The honeybee orthologue GB13602 has a Trp at the equivalent position, which should confer an insecticide susceptible phenotype.

Another corollary of the probable lack of carboxylesterase activity of the three honeybee enzymes with nonconsensus oxyanion holes is that it further reduces the number of *A. mellifera* esterase isozymes that are likely to be visualized after native polyacrylamide gel electrophoresis and staining with carboxylester substrates. Coupled with the fact that many of the CCEs in the other two classes are either catalytically inactive or likely to have very restricted expression profiles, we would predict a relative paucity of such isozymes in the honeybee. In fact just six have been recorded (Bitondi & Mestriner, 1983), about one-sixth as many as seen in *D. melanogaster* (Healy *et al*., 1991) and about one-tenth the number seen in some Lepidoptera (Oakeshott *et al*., 2005a).

## Discussion

Overall, there are significantly fewer genes in all three superfamilies in the honeybee than the other two species. However, the degree of difference is highly uneven across major clades within these superfamilies. This pattern suggests that the overall shortfall is not due to some general loss of diversity due to the haplodiploid genetic system of the bee. Instead it suggests much more specific tailoring of genome content to meet the needs of the organism. It is unclear at this point whether it generally reflects a loss of genes or a relative rarity of gene radiation events in the honeybee lineage. More genome data for other hymenopterans variously related to *A. mellifera* will be needed to resolve this question.

Where there are sufficient functional data available, it seems that many of the genes in the three superfamilies that have been retained in the honeybee are not involved in detoxification, so in relative terms the paucity of these genes in the honeybee is even greater in clades providing detoxification functions. It is at the least a parsimonious interpretation that the honeybee has less molecular flexibility in its scope for environmental response, although it is not at all clear just how many detoxification genes are necessary to provide adequate flexibility.

It is also a parsimonious interpretation that the deficit of detoxification genes in the honeybee will translate to less pesticide detoxification capability, which would then explain the species’ unusual sensitivity to pesticides. While it is tempting also to ascribe the absence of metabolic resistance to the shortage of detoxification genes, it must also be noted that target site resistance has not been reported either, so some of this might simply be explained by relatively infrequent exposure of reproductive individuals to the insecticides.

It has been suggested that the haplodiploid genetic system of honeybees may also contribute to reduced genetic diversity specifically related to xenobiotic detoxifying systems. Most of the theory relates to the fate of allelic variation, specifically the response to selection for resistance (Hartl, 1972; Denholm *et al*., 1998), rather than the extent of paralogous diversity within gene families, and even then there are differing predictions under different models (Carriere, 2003). Such empirical evidence is available (Theiling & Croft, 1988; Hoy, 1990) that pesticide susceptibility is generally higher, and resistance development slower, in parasitoids (which are generally haplodiploid) than in predators and pests (which are generally diploid). However, the implications of this theory in terms of the evolution of paralogous diversity in gene families are not clear.

Alternatively, the species’ highly organized eusociality may be part of the explanation for the low number of detoxifying genes in the genome. Because of this eusociality, which is further developed or more specialized than that of other eusocial Hymenoptera, the honeybee is capable of acting collectively to achieve a remarkable level of environmental homeostasis within the nest (Schmickl & Crailsheim, 2004). Reproductive individuals in particular are therefore less exposed to environmental stresses, including xenobiotics. One prediction from this explanation would be that the relevant gene clades would be larger in other apids that are not so highly eusocial.

We know of no data as yet that bear directly on this but the relevant clades of the phylogeny for the family Apidae are quite well established and dated by fossil and biogeographical data (Seeley, 1985; Ruttner, 1988; Moritz, 1992; Michener, 2000) and should lend themselves to some informative comparisons. Eusociality and some other behavioural adaptations that mitigate environmental exposure such as building nest covers and clustering of bees for nest protection are estimated to have arisen with the emergence of the subfamilies Meliponinae and Apinae (*Apis*) at least ≈ 80 Mya. However, the further development of cavity multicomb nesting, thermoregulatory behaviour (homeothermy) and the elaboration of a dance language for communication within the colony post-dates the divergence of the *A. cerana-mellifera* lineage from those including *A. florea*, *A*. *dorsata* and *A*. *laboriosa*, which first occur in the fossil record ≈ 22–25 Mya. The subsequent evolution of these adaptations then enabled the expansion of honeybees from tropical through to sub-Arctic environments and the radiation of the *A. cerana-mellifera* species group. Estimates of dates for this latter radiation vary from 1 to 2 out to 6 Mya.

One of the anomalies emerging from our analyses is that the chemical communication that underpins eusociality might itself require an amplification of some P450 and possibly CCE clades that are involved in pheromone biosynthesis and signal reception. In fact, we see only two or three instances of very recent radiations in any of the three superfamilies. The most obvious radiations that might represent such a process involve the CYP6s and CYP9s, although there is little functional information available as yet to implicate them directly in semiochemical biochemistry. One prediction of this proposition is that these radiations have occurred in the same relatively recent time frame as the species’ highly advanced eusociality has evolved.

As noted earlier, sequences involved in some paralogous comparisons within the CYP6 and CYP9 radiations (Fig. 2) show relatively little sequence divergence; silent site differences in many pair-wise comparisons within these radiations give small K_s_ values, of the order of 0.135–0.235. By comparison, terminal bifurcations in the radiation of CCEs in clade A (GB13591–GB15030 and GB19866–10854, Fig. 4) yield average numbers of changes per silent site in excess of 2.2, suggesting that their radiation is much older. Recent studies by Bromham & Leys (2005) show that the rate of silent site change is faster in the most highly eusocial Hymenoptera such as *A. mellifera* than it is in other species. There is as yet too little appropriate data to measure evolutionary rates in *A. florea, A. dorsata* or *A. laboriosa* compared with *A. mellifera*, but we note that the median silent site divergence between (the nonsocial) *D. melanogaster* and *Drosophila pseudoobscura*, which diverged 25–55 Mya, is 1.8 changes per site (Lemos *et al*., 2005; Richards *et al*., 2005). It therefore seems reasonable to propose that a good proportion of the duplication events in the two CYP radiations have evolved in the *A. cerana-mellifera* group as they split from other honeybee lineages and in the same time frame as their acquisition of more specialized eusocial and behavioural modes. By contrast, the CCE duplications in clade A are considerably older, possibly in the same time frame as the first emergence of eusociality at about ≈ 80 Mya.

Putting a time frame to the changes to the complements of these and other protein superfamilies down the *A. mellifera* lineage will be of fundamental importance, not just because it bears on the genetic architecture underlying the species’ semiochemical signalling, but also because it goes to the question of the evolutionary future for *A. mellifera*. Has its genome been pared down to such an extent as it has evolved such a highly specialized life-style that it now has limited flexibility to adapt to environmental changes outside its control? Does a multigene family version of Muller's ratchet apply in so much as the limited number of paralogous gene options in the honeybee is too few to mount a timely selective response to environmental change? Or is its streamlined genome simply the norm through much of the Hymenoptera, which overall have been highly successful in diversifying to exploit a wide range of environments? Further data on other *Apis* species and genome sequencing work now underway on *Nasonia* species will go a long way toward answering these questions.

Finally, in an applied context, we note that the honeybee is one of the key nontarget species considered in toxicological studies on new chemical and biological insecticides. Having learned that its complement of potential detoxifying systems is so limited it is now important to determine which of its residual GSTs, P450s and CCEs have the requisite expression and substrate specificities to provide xenobiotic detoxifying functions. Heterologous expression of this very small set of enzymes could provide a valuable resource for rational insecticide discovery and design. For example, it could be used to screen candidate new insecticides for molecules that honeybees will have relatively little difficulty metabolizing, or to identify pro-insecticides that bees would have little ability to activate.

## Experimental procedures

Sequences encoding GSTs, P450s and CCEs were identified using previously annotated *D. melanogaster* and *An. gambiae* proteins to TBLASTN (Altschul *et al*., 1997) search assembly Version 3.0 and 4.0 of the Honeybee Genome Project at the Human Genome Sequencing Centre, Baylor College of Medicine (http://www.hgsc.bcm.tmc.edu/). High probability hits were compared with Glean-3 predictions in BeeBase (http://racerx00.tamu.edu/bee_resources.html), and gene predictions at NCBI (http://www.ncbi.nlm.nih.gov/). Coding sequences were reconciled using available EST cDNA data held at The Institute for Genomic Research (TIGR) and the recently completed RIKEN honeybee cDNA database (searches conducted by Ryszard Maleszka). For genes without EST support, exon/intron boundaries were identified on the basis of TBLASTX alignments between selected portions of the honeybee genome and previously characterized/annotated sequences from other organisms. GeneSplicer splice site prediction software (Pertea *et al*., 2001) and BioPerl scripts (Stajich *et al*., 2002) also aided annotations. Putative amino acid sequences were aligned in a three-way genome comparison with previously reported *D. melanogaster* and *An. gambiae* sequences (Feyereisen, 2005; Ranson & Hemingway, 2005; Oakeshott *et al*., 2005a) using Clustal W. Unless otherwise specified, evolutionary distances were calculated from protein sequence data (standard amino acid code) using the proportional distance model with MEGA 3.1 (Kumar *et al*., 2004). Phylogenetic trees were determined by the neighbour-joining method with bootstrap resampling statistics (detailed in the legends for Figs 1, 2 and 4). Silent site nucleotide comparisons were calculated using DNAsp Version 4 (Rozas *et al*., 2003).

## Website references

http://P450.antibes.inra.fr

http://www.tigr.org

http://www.hgsc.bcm.tmc.edu

http://racerx00.tamu.edu/bee_resources.html

http://www.ncbi.nlm.nih.gov
